# Functional and Radiological Outcomes of Displaced Proximal Humerus Fractures Treated With the Proximal Humerus Internal Locking System (PHILOS) Plate: A Prospective Single-Center Observational Study

**DOI:** 10.7759/cureus.114192

**Published:** 2026-08-09

**Authors:** Harpreet Singh, Sangam Tyagi, Chandresh Choudhary, Mayank Kumar

**Affiliations:** 1 Orthopedics, Geetanjali Medical College & Hospital, Udaipur, IND

**Keywords:** constant–murley score, functional outcome, internal fixation, locking plate, philos plate, proximal humerus fracture

## Abstract

Background

Angular-stable fixation with the proximal humerus internal locking system (PHILOS) is widely used for displaced proximal humerus fractures. We prospectively evaluated its functional and radiological outcomes over 24 weeks at a tertiary-care center.

Methodology

Forty-three adults with proximal humerus fractures considered to require operative fixation underwent open reduction and internal fixation with a PHILOS plate. Pain (visual analog scale, VAS) and shoulder function (Constant-Murley score, CMS) were assessed at two, six, 12, and 24 weeks; time to union and immediate post-operative neck-shaft angle (NSA) and humeral head height were recorded. Serial change was analyzed with the Friedman test and, as confirmation, Greenhouse-Geisser-corrected repeated-measures analysis of variance (ANOVA), with Bonferroni-adjusted Wilcoxon post-hoc comparisons.

Results

Mean age was 48.0 ± 14.4 years (32 men, 11 women); all 43 patients completed follow-up. Mean total CMS rose from 40.7 ± 1.7 at 2 weeks to 54.3 ± 2.6, 66.6 ± 3.3 and 76.1 ± 3.6 at 6, 12 and 24 weeks (Friedman χ² = 129.0, p < 0.001; repeated-measures ANOVA p < 0.001). Mean VAS fell from 9.1 ± 0.6 pre-operatively to 1.2 ± 0.4 at 24 weeks (p < 0.001); all pairwise interval comparisons were significant (p < 0.001). At 24 weeks, 18.6% of shoulders were graded excellent, 79.1% good, and 2.3% fair. All fractures united (mean 9.8 ± 2.6 weeks). Final CMS was lower with higher Neer grade (p = 0.007) and increasing age (ρ = -0.35, p = 0.02). Immediate post-operative NSA (133 ± 3°) and head height (8.7 ± 1.3 mm) were within accepted limits.

Conclusions

PHILOS fixation produced progressive, statistically significant improvements in pain and shoulder function, with a 100% union rate over 24 weeks; outcomes were poorer in older patients and in patients with more complex fractures. The single-center design, the absence of a comparison group, and the mid-term follow-up limit generalisability, and longer follow-up is needed to capture late complications such as avascular necrosis.

## Introduction

Proximal humerus fractures are among the most common fractures of the upper limb and show a bimodal distribution, occurring as high-energy injuries in younger adults and as fragility fractures in older patients [1,2]. Most are managed non-operatively, but displaced and unstable patterns-particularly two-, three- and four-part fractures by the Neer classification-frequently require operative stabilization to restore anatomy and shoulder function [2].

Angular-stable locking plates are now a mainstay of fixation. The proximal humerus internal locking system (PHILOS) construct provides a fixed-angle design with divergent locking screws in the humeral head, improving purchase in osteoporotic and comminuted bone and permitting early rehabilitation [3,4]. Reported outcomes are generally favorable, but complications including varus collapse, screw cut-out and avascular necrosis remain important, particularly in complex fractures and older patients [5,6].

Although the literature is large, many series report only endpoint results or pre-operative versus final scores, with few providing evenly spaced longitudinal data on the early recovery trajectory. We therefore evaluated the clinical, functional, and radiological outcomes of PHILOS fixation using serial visual analog scale (VAS) and Constant-Murley assessments at two, six, 12, and 24 weeks, and examined the influence of fracture pattern and age on final function.

## Materials and methods

This prospective observational study was conducted in the Department of Orthopedics, Geetanjali Medical College and Hospital, Udaipur. The study period was 18 months from April 2024 to November 2025. Institutional ethics committee approval was obtained, and written informed consent was obtained from each participant. Reporting follows the strengthening the reporting of observational studies in epidemiology (STROBE) statement for observational studies [7].

Participants

Adults (>18 years) with fresh (<3 weeks), closed proximal humerus fractures considered to require operative fixation were eligible. The decision to operate, including for a small number of minimally displaced fractures, was made by the treating surgeon based on displacement, instability, and patient factors.

Exclusion criteria were compound injuries, pathological or malignancy-related fractures, established infection, ipsilateral clavicle/scapula fracture, distal neurovascular deficit, pre-existing shoulder pathology, and refusal of consent.

All eligible patients presenting during the study period were enrolled consecutively; no formal a priori power calculation was performed for the longitudinal outcome analysis, and the sample represents a convenience series of 43 patients.

Surgical technique

Fractures were classified by Neer criteria on Grashey and axillary/Velpeau radiographs, with CT where required [8]. Under general anesthesia in the beach-chair position, fixation was performed through a deltopectoral or minimally invasive deltoid-splitting approach. After provisional K-wire reduction, a PHILOS plate was applied with proximal locking and distal shaft screws; reduction and implant position were confirmed under image intensification. Antibiotic prophylaxis was given perioperatively.

Post-operative care and follow-up

The limb was supported in a sling for approximately six weeks with early elbow, wrist and hand mobilization, followed by progressive shoulder rehabilitation. Patients were reviewed at two, six, 12 and 24 weeks, and all 43 completed the 24-week assessment.

Outcome measures

The primary outcomes were pain on the 10-point VAS and shoulder function on the 100-point Constant-Murley shoulder score (CMS), recorded at each visit [9].

Radiological outcomes were time to union (bridging of ≥3 of 4 cortices) and the immediate post-operative neck-shaft angle and humeral head height. Patients were monitored clinically and radiographically for complications, including avascular necrosis, screw cut-out or penetration, loss of reduction, non-union and infection.

Statistical analysis

Data were analyzed in Python (SciPy). Categorical variables are presented as frequencies and percentages, continuous variables as mean ± standard deviation. Serial change in CMS and VAS was assessed with the non-parametric Friedman test (Kendall's W as effect size) and, as a parametric confirmation, one-way repeated-measures ANOVA with Greenhouse-Geisser correction for sphericity (partial η² as effect size); because VAS is ordinal and non-normally distributed, the Friedman test was regarded as primary for pain. Pairwise post-hoc comparisons used the Wilcoxon signed-rank test with Bonferroni adjustment. Associations between final CMS and Neer type and age were examined using the Kruskal-Wallis test and Spearman's correlation. Two-sided p < 0.05 was significant.

## Results

Baseline characteristics

Forty-three patients (32 men, 11 women; mean age 48.0 ± 14.4 years, range 28-74) were analyzed, with complete 24-week follow-up. The most common mechanism was a fall on the outstretched hand (22, 51.2%), followed by road traffic accidents (19, 44.2%) and assault (2, 4.7%). Left and right shoulders were affected in 23 (53.5%) and 20 (46.5%), respectively. Twenty-one patients (48.8%) had at least one comorbidity, most commonly hypertension. Baseline and fracture characteristics are shown in Tables 1 and 2.

**Table 1:** Baseline demographic and clinical characteristics (n = 43) Mean age 48.0 ± 14.4 years. Percentages may not total 100 owing to rounding.

Characteristic	Category	n (%)
Age group (years)	≤30	2 (4.7)
	31-45	18 (41.9)
	46-60	12 (27.9)
	61-75	11 (25.6)
Sex	Male	32 (74.4)
	Female	11 (25.6)
Mechanism	Fall on outstretched hand	22 (51.2)
	Road-traffic accident	19 (44.2)
	Assault	2 (4.7)
Side	Left	23 (53.5)
	Right	20 (46.5)
Comorbidity	None	22 (51.2)
	Hypertension (± asthma)	12 (27.9)
	Diabetes mellitus	7 (16.3)
	Asthma only	2 (4.7)

**Table 2:** Fracture pattern and peri-operative variables (n = 43) Six minimally displaced (Neer one-part) fractures were managed operatively at the treating surgeon's discretion

Variable	Category	Value
Neer classification	Two-part	23 (53.5%)
	Three-part	11 (25.6%)
	One-part (minimally displaced)	6 (14.0%)
	Four-part	3 (7.0%)
Hospital stay (days)	Mean (range)	3.1 (3-5)
Admission-to-surgery (days)	Mean (range)	1.9 (1-3)
Pre-operative VAS	Mean ± SD	9.1 ± 0.6

Pain and functional recovery

**Figure 1:**
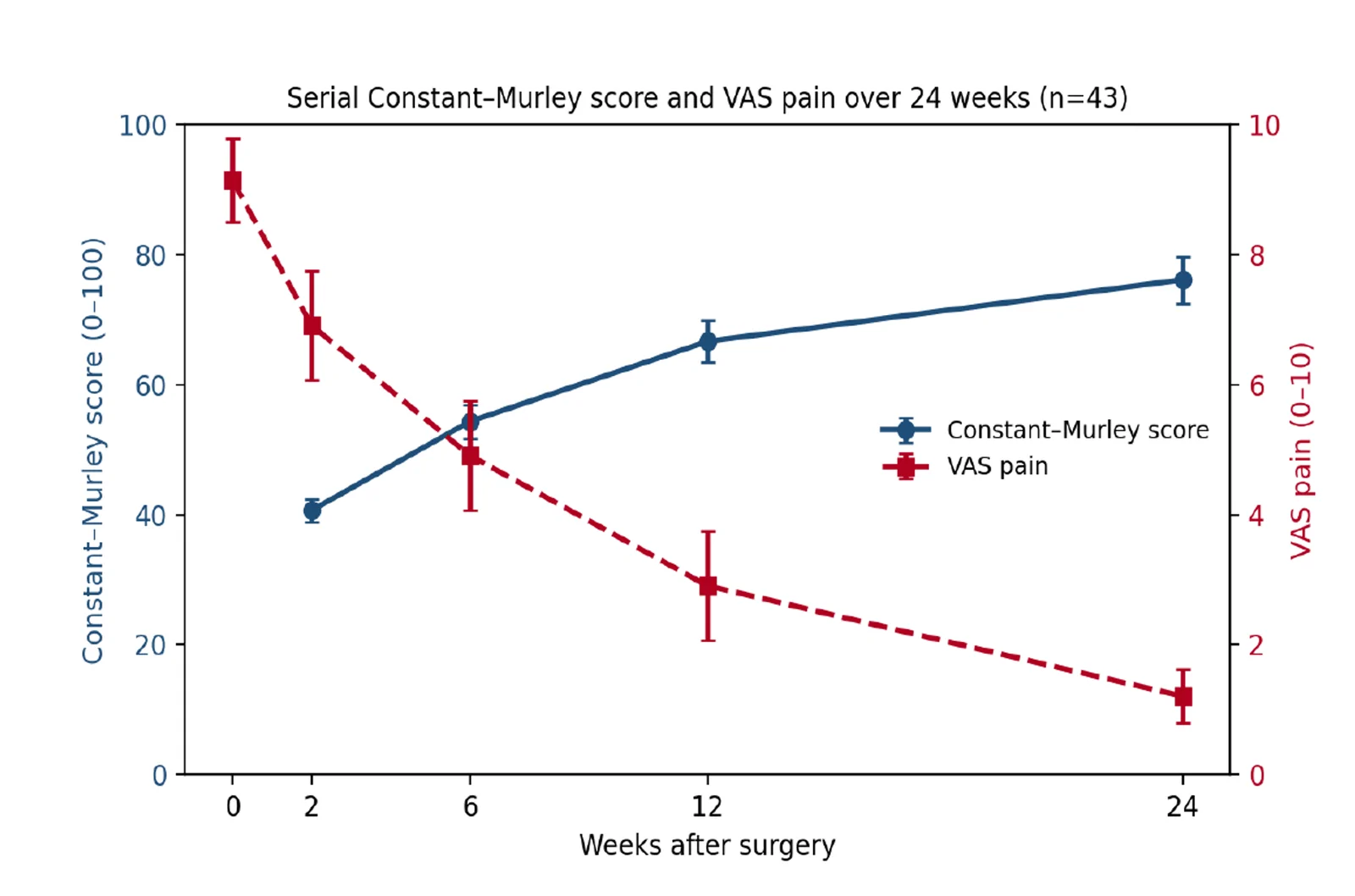
Serial mean Constant-Murley score and VAS pain over 24 weeks (error bars = SD)

Pain decreased progressively from a mean pre-operative VAS of 9.1 ± 0.6 to 6.9 ± 0.8, 4.9 ± 0.8, 2.9 ± 0.8 and 1.2 ± 0.4 at two, six, 12 and 24 weeks. Mean total CMS rose from 40.7 ± 1.7 at 2 weeks to 76.1 ± 3.6 at 24 weeks, a mean gain of 35.4 points. Both trajectories were highly significant on the Friedman test (VAS χ² = 128.2; CMS χ² = 129.0; both p < 0.001), and on Greenhouse-Geisser-corrected repeated-measures ANOVA (both p < 0.001), and every pairwise interval comparison remained significant after Bonferroni adjustment (all p < 0.001). Because all patients improved monotonically, within-subject effect sizes were expectedly large (partial η² 0.97-0.99) and should be read as reflecting universal recovery over time rather than a between-group treatment effect. Serial values are given in Table 3 and Figure 1.

**Table 3:** Serial VAS pain and total Constant-Murley scores (mean ± SD) across the four post-operative time points Pre-operative VAS 9.1 ± 0.6. Greenhouse-Geisser-corrected repeated-measures ANOVA also p < 0.001 for both outcomes; all Bonferroni-adjusted pairwise comparisons p < 0.001.

Outcome	2 weeks	6 weeks	12 weeks	24 weeks	p (Friedman)
VAS pain (0-10)	6.9 ± 0.8	4.9 ± 0.8	2.9 ± 0.8	1.2 ± 0.4	<0.001
Constant-Murley (0-100)	40.7 ± 1.7	54.3 ± 2.6	66.6 ± 3.3	76.1 ± 3.6	<0.001

Functional grade

Using a categorical interpretation of the Constant-Murley score (excellent ≥80, good 70-79, fair 60-69, poor <60), 8 shoulders (18.6%) were excellent, 34 (79.1%) good and 1 (2.3%) fair at 24 weeks; none were poor (Table 4). As categorical banding of the Constant score is not standardized, the continuous score (Table 3) is the primary functional outcome.

**Table 4:** Constant-Murley functional grade at 24 weeks (n = 43)

Grade	n	%
Excellent (≥80)	8	18.6
Good (70-79)	34	79.1
Fair (60-69)	1	2.3
Poor (<60)	0	0

Radiological outcomes

All 43 fractures achieved radiological union, at a mean of 9.8 ± 2.6 weeks (range 6-15): within 6-8 weeks in 13 (30.2%), 9-12 weeks in 20 (46.5%) and 13-15 weeks in 10 (23.3%). The immediate post-operative neck-shaft angle (mean 133 ± 3°, range 128-139°) lay within the normal 130-140° range, and humeral head height (mean 8.7 ± 1.3 mm, range 6-10) was within accepted limits (Table 5), consistent with adequate reduction. Serial radiographic measurements over follow-up were not available in the analyzed dataset.

**Table 5:** Radiological outcomes (n = 43)

Parameter	Value
Union rate	43/43
Mean time to union	9.8 ± 2.6 weeks
Union 6-8 / 9-12 / 13-15 weeks	13 / 20 / 10
Neck-shaft angle (immediate post-op)	133 ± 3°
Humeral head height (immediate post-op)	8.7 ± 1.3 mm

Factors associated with outcome. Final CMS differed significantly by Neer type (Kruskal-Wallis p = 0.007): mean 24-week scores were 77.5 ± 2.8 (two-part), 77.0 ± 3.9 (one-part), 73.9 ± 3.4 (three-part) and 71.7 ± 2.9 (four-part). Increasing age was associated with a lower final score (Spearman ρ = −0.35, p = 0.02), consistent with poorer recovery in older patients and more comminuted fractures.

Complications

No implant-related complication-avascular necrosis, screw cut-out or penetration, loss of reduction, non-union, or deep infection-was documented during the 24-week follow-up.

## Discussion

In this prospective series of 43 displaced proximal humerus fractures treated with PHILOS fixation, pain and shoulder function improved progressively and significantly over 24 weeks, with a mean final Constant-Murley score of 76 and a 100% union rate. The evenly spaced assessments at two, six, 12 and 24 weeks characterized the recovery trajectory itself, showing the largest gains between two and 12 weeks, followed by continued but slower improvement to 24 weeks.

These findings are consistent with contemporary locking-plate series. Rathod et al. and Narasimha Rao et al. reported comparable stepwise Constant-score improvement after PHILOS fixation, and Kugashiya et al. described similar functional gains across Neer types, although absolute values differ with follow-up length and case mix [10-12]. As expected from the underlying biology, final function was poorer with higher Neer grade and older age, echoing reports that comminution and osteoporosis are associated with less complete recovery and higher complication rates [13,14].

Immediate post-operative alignment was within accepted limits, with a mean neck-shaft angle of 133°. This is relevant because varus malreduction and loss of medial support are established predictors of fixation failure and reduction loss after locking-plate fixation [15-17]. However, serial radiographic follow-up was not analyzed in the present dataset, so conclusions about late loss of reduction cannot be drawn.

The absence of documented early complications must be interpreted cautiously. Systematic reviews and meta-analyses report clinically important complications and reintervention rates after PHILOS fixation, and avascular necrosis often becomes apparent only beyond six months [5,18,19]. A 24-week window is therefore insufficient to exclude these events, and the present findings reflect only the early post-operative period.

This study has strengths: a prospective design, standardized surgical technique and rehabilitation, validated outcome instruments, complete 24-week follow-up and formal repeated-measures analysis [20]. It also has important limitations. The sample is modest, drawn from a single center and not formally powered, limiting generalisability. Follow-up extends only to 24 weeks - too short to capture late complications or the durability of function - and there was no comparison group or patient-reported quality-of-life measure. Functional scoring commenced at two weeks, so the absence of a pre-operative or immediate post-operative Constant-Murley baseline precludes quantification of change from the injured state. Six minimally displaced (Neer one-part) fractures were managed operatively, which deviates from a strict displaced-only criterion and may modestly favor the aggregate outcome. Finally, the Constant-score grade bands are not standardized, and only immediate post-operative radiological measurements were available, so the continuous functional scores should be regarded as the principal result.

## Conclusions

PHILOS plating for displaced proximal humerus fractures produced progressive, statistically significant improvement in pain and shoulder function, a 100% union rate and acceptable immediate post-operative alignment over 24 weeks, with most patients achieving good functional outcomes. Recovery was poorer in older patients and more complex fracture patterns. Within the constraints of a single-center, non-comparative design and mid-term follow-up, PHILOS fixation is a reliable option for appropriately selected patients; longer-term comparative studies are needed to define durability and late-complication rates.

## Figures and Tables

**Figure 2:**
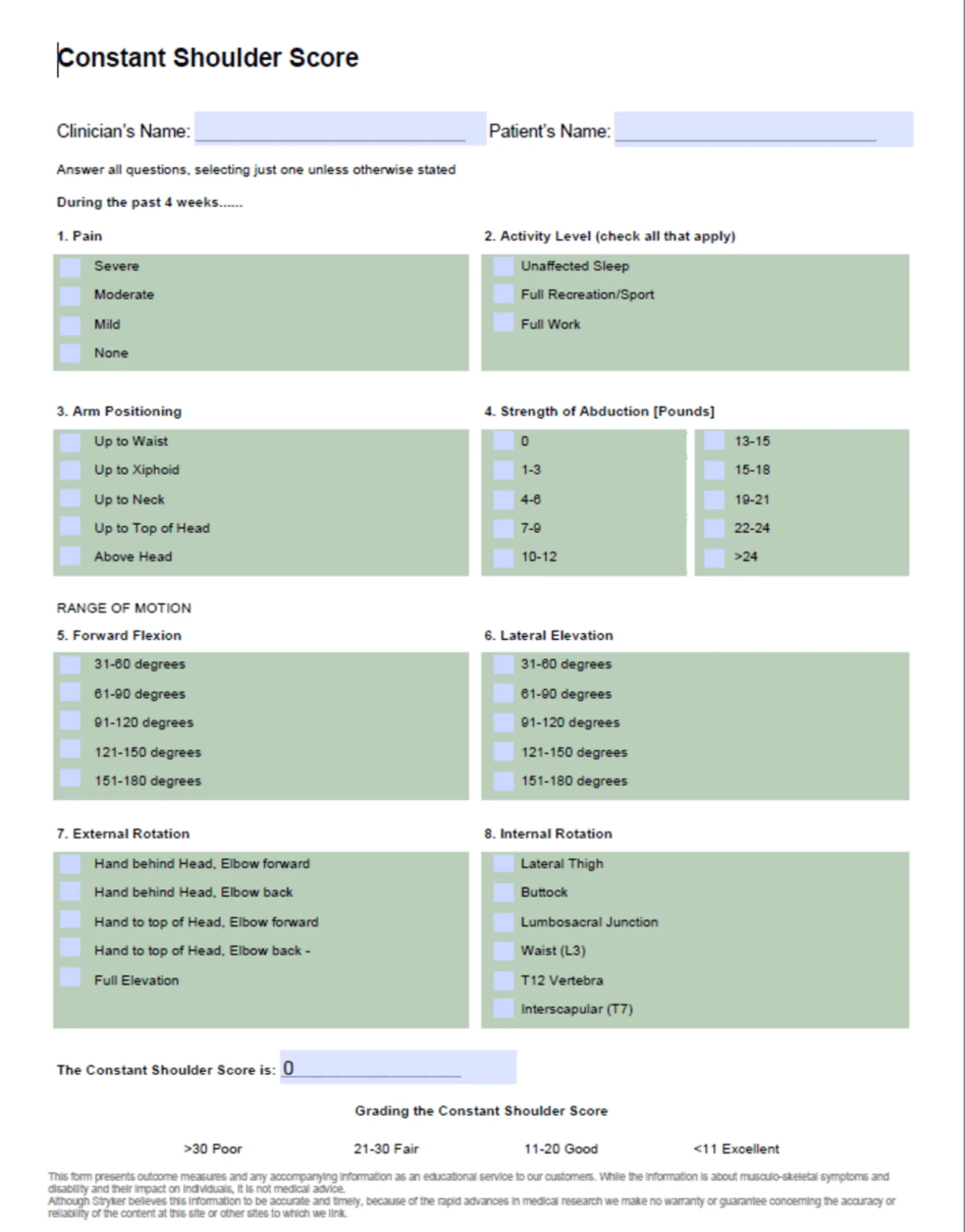
Constant-Murley Shoulder score

## Appendices

**Table 6:** Informed consent form

Informed consent form	
Title of study	Functional and Radiological Outcomes of Displaced Proximal Humerus Fractures Treated with the Proximal Humerus Internal Locking System (PHILOS) Plate: A Prospective Single-Centre Observational Study
Name of Principal Investigator:	Dr Chandresh Choudhary
Name of Organisation	Geetanjali University
Name of Moderator	Dr Harpreet Singh

**Table 7:** Part I: Information Sheet, Part II: Certificate of consent, Part III: Statement by the researcher

PART I: Information Sheet	
Introduction	I am Dr CHANDRESH CHOUDHARY, working for the Orthopaedic Department of Geetanjali University. We are researching “FUNCTIONAL OUTCOMES OF THE PROXIMAL HUMERUS FRACTURE TREATED SURGICALLY WITH PHILOS PLATE”. I will provide information about our research and invite you to be part of it. You can take as much time as you want and talk with anyone you feel comfortable with before deciding if you want to participate in this research. If at any point you have questions, please ask me to go through it, and I will explain.
Purpose of the research	To study the Functional and Radiological Outcomes of Displaced Proximal Humerus Fractures Treated with the PHILOS Plate.
Participant selection	We invite all patients aged> 18 years with proximal humerus fractures to attend the Orthopaedic Department at Geetanjali Hospital for treatment. Patients will be taken up for internal fixation, i.e., PHILOS plating, via the Deltopectoral or minimally invasive Deltoid-splitting approach.
Voluntary Participation	Your participation in the study is completely voluntary, and you may refuse to answer any questions or choose to stop participating at any time. Whether you choose to participate or not, all the services you receive at this clinic will continue, and nothing will change. You can stop participating in the study at any time, for any reason, if you so decide. If you decide to stop participating, it will not influence the treatment you may be receiving either now or in the future.
Procedures and Protocol	Patients with Proximal Humerus Fracture will be treated surgically with Philos Plating, after full clinical and radiological assessment, and will be assessed functionally postoperatively to determine treatment outcomes. If there is anything you are concerned about or that is bothering you about the research, please talk to one of the researchers or me.
Confidentiality	All information you provide during the research will be kept confidential. Unless you specifically indicate your consent, your name will not appear in any report or publication of the research. Confidentiality will be provided to the fullest extent possible by law.
Questions about the research	If you have any questions about the research in general or about your role in the study, please feel free to contact: Dr. CHANDRESH CHOUDHARY Post-Graduate Resident Department of Orthopaedics, GMCH, Udaipur, E-mail: chandresh91995@gmail.com
PART II: Certificate of Consent	
I have read the foregoing information, or it has been read to me. I have had the opportunity to ask questions about it, and any questions that I have asked have been answered to my satisfaction. I voluntarily consent to participate in this research.	Print Name of Participant Signature of Participant Date
If illiterate – A literate witness must sign (if possible, this person should be selected by the participant and should have no connection to the research team). Illiterate participants should also include their thumbprint. I have witnessed the accurate reading of the consent form to the potential participant, and the individual has had the opportunity to ask questions. I confirm that the individual has given consent freely.	A Print name of the witness Thumbprint of participant. Signature of witness Date
PART III: Statement by the researcher.	
I have accurately read the information sheet to the potential participant and, to the best of my ability, ensured that the participant understands what will be done and the associated risks and benefits. I confirm that the participant was allowed to ask questions about the study, and that all questions asked have been answered correctly, to the best of my ability. I confirm that the individual has not been coerced into giving consent, and that the consent was given freely and voluntarily.	A copy of this ICF has been provided to the participant. Print Name of Researcher/person taking the consent Signature of Researcher / person taking the consent Date

**Table 8:** Case record proforma

Case record proforma
Inpatient no.
Age/gender
Occupation
Mechanism of injury
Neer fracture
Shoulder site duration of stay
Days to surgery
Pre-op VAS
Comorbidities
Follow-up
VAS follow-up
VAS follow-up (2nd week)
VAS follow-up (6th week)
VAS follow-up (12th week)
VAS follow-up (24th week)
CMS follow-up
CMS score (2nd week)
CMS score (6th week)
CMS score (12th week)
CMS score (24th week)
Radiological outcome
Union time (week)
Neck-shaft angle (degrees)
Humeral head height (mm)
